# A systematic pipeline for diagnosing and reducing gender-stereotype bias in Japanese PLMs for sentiment analysis

**DOI:** 10.3389/frai.2026.1851069

**Published:** 2026-06-03

**Authors:** Yang Liu, Ziyang Li

**Affiliations:** 1 College of Eastern Language and Culture, Harbin Normal University, Heilongjiang, Harbin, China; 2 College of Art, Northeast Agricultural University, Heilongjiang, Harbin, China

**Keywords:** algorithmic fairness, bias mitigation, gender bias, japanese NLP, pre-trained language models, sentiment analysis

## Abstract

Pre-trained language models (PLMs) are widely used in sentiment analysis, but they may inherit gender-stereotypical bias from large-scale text corpora and transfer such bias to downstream sentiment predictions. Despite growing attention to gender-stereotypical bias in PLMs, existing studies predominantly focus on English corpora and static word embeddings, limiting understanding of how such bias affects sentiment analysis models and the effectiveness of mitigation strategies. In this study, we present a three-stage task-oriented pipeline for diagnosing, mitigating, and evaluating gender-stereotypical bias in Japanese PLMs for sentiment analysis tasks. Specifically, the proposed framework diagnoses bias based on pro-stereotypical (PS), anti-stereotypical (AS), and neutral test sets, which are constructed to compare stereotype-aligned, stereotype-violating, and gender-neutral contexts under the same sentiment analysis setting. We further introduce two complementary evaluation measures, the Stereotype Bias Index (SBI) and Gender Sentiment Bias (GSB), to quantify stereotype-level bias between PS and AS samples, as well as sentiment prediction differences among male, female, and neutral groups. To mitigate bias, the framework performs debiasing fine-tuning using gender-swapped training data and then quantitatively evaluates bias reduction while monitoring sentiment classification performance. Experimental results on three Japanese BERT-based sentiment analysis models demonstrate that the proposed pipeline substantially reduces gender-stereotypical bias. For the SBI metric, the bias magnitude is reduced by 97.4%, 70.0%, and 76.9% for Tohoku BERT_BASE_, Tohoku BERT_BASE_(chABSA), and Tohoku BERT_BASE_(JSPD), respectively. For the GSB metric, the bias magnitude is consistently reduced across gender-group comparisons, with reduction rates ranging from 33.3% to 98.8%. Meanwhile, sentiment classification performance is maintained or slightly improved after debiasing.

## 1 Introduction

Pre-trained language models (PLMs) learn linguistic patterns and semantic representations from text corpora and have become a fundamental cornerstone in natural language processing (NLP), providing strong support for a wide range of downstream tasks such as machine translation, question answering, and particularly sentiment analysis (Somayajula et al., 2024; Wang et al., 2023; Hu et al., 2024). Sentiment analysis, which aims to identify and quantify the emotional polarity expressed in text, plays a crucial role in areas such as social media monitoring, product analysis, and workforce analytics (Abdullah and Ahmet, 2022). Its effectiveness largely depends on the quality and neutrality of the linguistic representations learned by PLMs.

However, existing evidence indicates that PLMs trained on large-scale text corpora inevitably inherit and reflect the social biases present in their training data (Fang et al., 2024; Jiang et al., 2025), which may be related to gender, race, age, or other attributes. Among these, gender-stereotypical bias is the most prevalent and socially scrutinized, and therefore, this study focuses specifically on gender-stereotypical bias in PLMs. For example, PLMs may disproportionately associate certain occupations, emotions, or adjectives with a specific gender-linking occupations such as *nurse* with females and *doctor* with males (Kotek et al., 2023), emotions such as *sadness* with females and *anger* with males (Plaza-del Arco et al., 2024). When such gender-stereotypical bias propagates to sentiment analysis models, it can lead to unfair predictions, thereby undermining the reliability and fairness of NLP applications (Jentzsch and Turan, 2022). Therefore, identifying, quantifying, and mitigating gender-stereotypical bias in PLMs is particularly important, especially in linguistically diverse and culturally specific contexts such as Japanese.

Japanese provides an important and challenging context for studying this problem. Although Japanese does not mark grammatical gender in the same way as some other languages, gender-related information can still be expressed through pronouns, first-person self-reference forms, role nouns, and culturally associated expressions (Sherwood et al., 2023). For instance, terms such as 彼 (he), 彼女 (she), 男性 (male), and 女性 (female), as well as self-reference forms such as 僕, 俺, and 私, may introduce explicit or implicit gender cues. In sentiment analysis, these cues can interact with occupation- or role-related expressions, such as 男性外科医 (male surgeon), 女性外科医 (female surgeon), 女性看護師 (female nurse), and 男性看護師 (male nurse). If two sentences express the same sentiment but differ only in gender-related terms, a fair sentiment analysis model should produce consistent predictions. However, PLMs may assign different sentiment polarities or confidence scores to such paired inputs, suggesting that gender cues rather than sentiment semantics influence model behavior.

Although attention to gender-stereotypical bias in PLMs has been increasing (Li et al., 2024; Zakizadeh and Pilehvar, 2025; Puttick et al., 2025), the specific gap addressed in this study remains insufficiently explored. Existing studies predominantly focus on English corpora, while research on languages with culturally nuanced gender expressions, such as Japanese, remains relatively limited (Puttick et al., 2025; Khandokar and Deshpande, 2026). Moreover, many existing bias measurement efforts focus primarily on static word embeddings or token-level representations (Caliskan et al., 2022; Ohamadike et al., 2025). However, such approaches do not directly capture how gender-stereotypical bias affects the final predictions of sentiment analysis models, thereby limiting our understanding of how bias propagates from linguistic representations to downstream sentiment decisions (Miranda e Silva et al., 2026). For Japanese sentiment analysis, it is still unclear how to construct controlled bias detection data, how to distinguish stereotype-level bias from broader gender-related sentiment differences, and how to evaluate whether a debiasing intervention reduces bias without degrading classification performance. Thus, the main research gap is not only the lack of Japanese bias studies, but also the absence of a task-oriented evaluation-and-mitigation pipeline for Japanese PLMs in sentiment analysis.

Motivated by these gaps, this study aims to develop a task-oriented framework for diagnosing, mitigating, and evaluating gender-stereotypical bias in Japanese PLMs for sentiment analysis tasks. Specifically, the framework first performs bias diagnosis on the PLM using a controlled bias detection dataset consisting of pro-stereotypical (PS), anti-stereotypical (AS), and neutral (NA) samples. The PS and AS samples are designed to compare stereotype-aligned and stereotype-violating contexts, while the NA samples provide gender-neutral baselines. Next, two complementary bias metrics are introduced: the Stereotype Bias Index (SBI), which quantifies bias between PS and AS contexts, and Gender Sentiment Bias (GSB), which measures sentiment prediction differences across male, female, and neutral groups. Finally, a subset of samples from the original data is selected and gender-swapped to create a debiasing training set, which is then used to fine-tune the model. The fine-tuned model is re-evaluated on the same bias detection data to quantify the degree of bias reduction and assess whether downstream sentiment classification performance is maintained. Therefore, the novelty of this study lies not merely in applying gender swapping to Japanese sentiment analysis, but in integrating controlled bias data construction, explicit bias metrics, debiasing fine-tuning, and post-debiasing evaluation into a unified task-level pipeline.

## 2 Related works

### 2.1 Bias evaluation in PLMs

Recent research has proposed a variety of methods to quantify social biases embedded in PLMs. For static word embeddings, Caliskan et al. (2017) proposed the WEAT score, which quantifies bias by comparing the relative similarity differences between two sets of target words and two sets of attribute words. For contextualized representations, researchers typically measure bias based on the probabilities of masked male or female word tokens, as well as unmasked tokens, in a given sentence using a masked language model (Kaneko et al., 2022). Beyond intrinsic evaluation, bias can also be assessed through a model's predictive behavior on downstream tasks. Dolci et al. (2023) proposed a bias score that estimates gender bias at the sentence embedding level by leveraging semantic word importance and gender-neutral information encoded in sentence embeddings. Park et al. (2023) proposed the stereotype quantification score to measure a model's consistency in gender pronoun prediction tasks. Anantaprayoon et al. (2024) evaluated gender discrimination in PLMs by constructing sentence pairs with gender perturbations and measuring the resulting changes in similarity scores. In natural language inference tasks for PLMs, Dev et al. (2020) proposed a method for evaluating bias by examining whether a model classifies sentence pairs such as *The doctor is writing a report* and *The man is writing a report* as neutral, thereby revealing potential gender-stereotypical bias. Anantaprayoon et al. (2024) introduced the NLI-CoAL bias evaluation method, which detects model biases by constructing three evaluation datasets representing different types of biases and defining corresponding bias metrics based on the label outputs associated with each dataset. Although these studies provide important tools for detecting bias in PLMs, their evaluation targets are not fully aligned with the present task. Embedding-based and token-probability-based measures mainly capture representational associations or local prediction preferences, but they do not directly indicate whether a sentiment analysis model produces different positive or negative predictions for stereotype-aligned and stereotype-violating contexts. Similarly, sentence-pair similarity or NLI-based metrics can reveal sensitivity to gender perturbations, but they are not specifically designed to measure sentiment classification disparities. Therefore, for Japanese sentiment analysis, a task-level metric is needed to quantify whether gender stereotypes affect the final sentiment predictions of PLMs. This motivates the Stereotype Bias Index (SBI), which directly compares class-level recall differences between pro-stereotypical (PS) and anti-stereotypical (AS) contexts.

Most existing bias evaluation studies primarily focus on English. However, due to the unique linguistic characteristics and cultural contexts of different languages, directly transferring bias detection methods from English to other languages is often unreliable. Research on Chinese and Japanese has shown that token-level or character-level representations can significantly affect the effectiveness of gender bias detection (Jiao and Luo, 2021). To address the specific features of different languages, researchers have developed various language-specific bias evaluation benchmarks and models, including CLUE for Chinese (Xu et al., 2020), KLUE for Korean (Park et al., 2021), and JBBQ for Japanese (Yanaka et al., 2025). Although language-specific bias evaluation resources, including Japanese benchmarks, have recently been developed, many of them are designed for general bias detection, question answering, or reasoning-oriented tasks rather than sentiment analysis. Consequently, they do not directly reveal whether gender-related cues alter predicted sentiment polarity or class-level recall. This issue is particularly important for Japanese PLMs, where gender cues may interact with culturally associated roles, occupations, and everyday expressions. Therefore, controlled sentiment analysis data are needed to distinguish stereotype-aligned, stereotype-violating, and gender-neutral contexts. This motivates our construction of PS, AS, and NA detection data and the introduction of SBI and GSB as task-level bias measures.

### 2.2 Bias mitigation in PLMs

**Figure 1:**
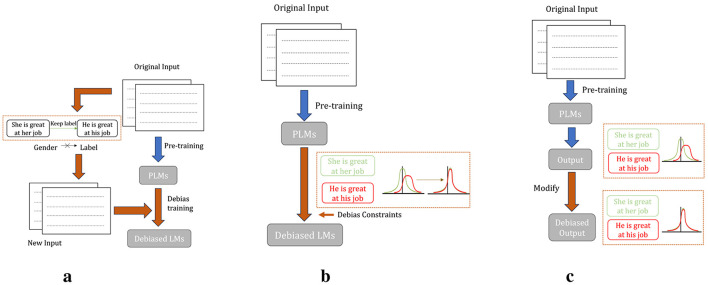
Brief illustration of existing bias debias methods for PLMs. **(a)** Pre-processing bias debias method. **(b)** In-processing bias debias method. **(c)** Post-processing bias debias method.

To reduce the inherent social biases in PLMs, researchers have proposed a variety of mitigation strategies at different levels, such as data augmentation, parameter-efficient tuning, and causal intervention. Depending on the stage at which the mitigation strategy is applied, these methods are typically categorized into three groups: pre-processing, in-processing, and post-processing. The workflows corresponding to these three categories are illustrated in Figure 1.

Pre-processing methods primarily mitigate bias by adjusting the model's input to weaken the correlation between demographic attributes and stereotypical associations, as shown in Figure 1a. Among them, a widely adopted approach is counterfactual data augmentation. Lu et al. (2020) propoesed to enhance the corpus through causal intervention by adding gender-matched paired samples to counteract gender bias in occupational domains, thereby weakening incorrect associations between gender and ostensibly neutral terms. Thakur et al. (2023) proposed to identify the most bias-revealing samples in a dataset and then applying data interventions by replacing gendered terms with more neutral or equality-oriented expressions. Bartl and Leavy (2024) further constructed a catalog of gender-exclusive English terms and gender-neutral variants, and used the resulting gender-inclusive fine-tuning corpus to reduce gender-stereotyping tendencies in LLMs. As shown in Figure 1b, in-processing methods aim to mitigate bias by adjusting the model training procedure, thereby reducing bias that arises during the training or fine-tuning of the model. Wang and Demberg (2024) introduced a multi-objective probability alignment approach during model training, which integrates multiple debiasing loss functions to identify and suppress various forms of bias. Xu et al. (2025) proposed the BiasEdit method, which employs model editing techniques to remove stereotypical biases from PLMs. As illustrated in Figure 1c, post-processing strategies aim to mitigate bias by adjusting the model's prediction outputs. Tokpo and Calders (2022) proposed a style transfer model that converts biased text into a more neutral version while preserving key semantic information and reducing bias in textual data. Dawkins et al. (2024) proposed projecting sentence representations into a neutral subspace to reduce their bias along the gender direction, without explicitly modifying the model parameters.

These studies show that bias can be mitigated at different stages of the PLM pipeline, but debiasing methods still require task-specific evaluation to verify their effects on downstream predictions. Although counterfactual data augmentation and gender swapping are widely used, their effectiveness may vary across languages and tasks. Therefore, for Japanese sentiment analysis, this study integrates fine-tuning on gender-swapped data with controlled bias diagnosis and post-debiasing evaluation to assess whether it reduces both stereotype-level and gender-group sentiment bias while preserving classification performance.

### 2.3 Bias in PLM-based sentiment analysis

In the domain of sentiment analysis, PLMs have been shown to exhibit systematic gender and racial biases when assigning sentiment polarity (Levy et al., 2023). Various methods have been proposed to detect bias in PLMs within the domain of sentiment analysis. Huang et al. (2020) employed a counterfactual evaluation approach to quantify sentiment biases using a fairness metric based on the Wasserstein distance, and further reduced these biases by applying regularization to the model's latent representations. Corizzo and Hafner (2024) proposed an ethnicity-aware algorithmic design approach to mitigate social biases in sentiment classification models. Liu et al. (2024) proposed the integrated gap gradients method to locate social bias neurons in PLMs that are associated with societal biases, and further introduced a bias neuron suppression mechanism to suppress these neurons, thereby enabling both the detection and mitigation of model bias. Radaideh et al. (2025) measured bias by comparing the distributions of model-predicted sentiment across different social groups and found that fine-tuning can effectively improve the model's fairness.

Compared with intrinsic bias evaluation, sentiment analysis requires a direct examination of prediction-level disparities, because the key concern is whether gender cues alter sentiment labels or class-level recall. Existing sentiment-bias measures often capture distributional differences, counterfactual shifts, or group-level disparities, but they do not explicitly distinguish stereotype-level bias between PS and AS contexts from broader gender-related sentiment bias among male, female, and neutral contexts. Therefore, this study introduces two complementary metrics: SBI for measuring PS-AS sentiment disparities and GSB for measuring gender-group sentiment differences. These metrics provide a more fine-grained basis for evaluating Japanese PLMs and assessing whether debiasing reduces bias while preserving downstream performance.

## 3 Materials and methods

### 3.1 Construction of bias detection data

To rigorously evaluate gender-stereotypical bias in Japanese PLMs for sentiment analysis tasks, we draw inspiration from previous research Anantaprayoon et al. (2024) and construct corresponding bias detection data for models pre-trained on different Japanese corpora. The bias detection data consists of three complementary categories: Pro-stereotypical (PS), Anti-stereotypical (AS), and Neutral (NA). The details of each category are described as follows.

#### 3.1.1 Pro-stereotypical (PS)

This category consists of sentences in which gender align with widely observed social stereotypes in Japanese society. Typical examples include occupational pairings such as 女性看護師 (female nurse) or 男性外科医 (male surgeon), which conform to common gender associations. In the construction process, examples were classified as PS when the gender-related expression in the original or generated sentence matched the predefined stereotype orientation assigned to the corresponding source expression. By presenting stereotype-consistent expressions, the PS set allows us to examine whether PLMs disproportionately favor sentiment predictions that implicitly reflect societal expectations.

#### 3.1.2 Anti-stereotypical (AS)

In contrast to PS, the AS category contains sentences that intentionally violate stereotypical gender associations, such as 男性看護師 (male nurse) or 女性外科医 (female surgeon). AS examples were generated by replacing the gender-related term in the corresponding PS expression with its counterpart while keeping the remaining sentence structure, semantic content, and original sentiment label unchanged. In this way, each AS example was designed to be comparable to its PS counterpart except for the gender cue. These samples are essential for detecting asymmetric model behavior: if a model shows divergent sentiment predictions between PS and AS instances with otherwise comparable semantics, such discrepancies indicate the presence of stereotype-driven bias within the learned representations.

#### 3.1.3 Neutral (NA)

This category includes sentences that either omit gender information entirely or adopt gender-neutral phrasings. Typical examples include purely neutral occupational terms such as 看護師 (nurse) or 外科医 (surgeon), as well as explicitly gender-neutral expressions like 医療従事者 (medical professional). NA examples were used as gender-neutral baselines and were not assigned PS or AS stereotype orientations. They were constructed from source expressions in which explicit gender cues were removed or replaced with neutral terms, while the sentiment label was preserved. By removing explicit gender cues, this category serves as a baseline that reflects unbiased linguistic conditions. Comparing NA with the PS and AS categories enables us to quantify the incremental effects introduced by gender-specific terms.

Together, these three categories form a controlled and comprehensive bias detection data for diagnosing gender stereotype bias. The construction of the bias detection data followed a controlled generation procedure. First, candidate source sentences were extracted from the corresponding sentiment datasets by identifying gender-related or stereotype-associated expressions, including gender terms, pronouns, self-reference forms, occupation-related terms, role nouns, adjectives, and activity-related descriptions. Second, source expressions associated with predefined gender-role stereotype categories in Japanese social and cultural contexts were selected and assigned stereotype labels. Third, matched PS and AS samples were constructed by varying the gender-related terms while keeping the remaining semantic content and the original sentiment label unchanged. NA samples were constructed by removing explicit gender cues or replacing gender-specific expressions with gender-neutral alternatives. The numbers of the resulting PS/AS and NA samples for each dataset used in this paper are reported in Table 1. Because bias detection data is strictly designed for evaluation, none of the items included in these categories are used during model training or hyperparameter tuning. This separation ensures that the bias measurements reflect inherent model behavior rather than artifacts of the training process.

**Table 1:** The description of bias detection dataset.

Dataset	Train	Val	Test	Classes	PS/AS	NA	Model
JSC	9,831	1,677	2,552	positive, negative	1,416	2,813	Tohoku BERT_BASE_ (chABSA), Tohoku BERT_BASE_ (JSPD)
MAR	200,000	5,000	5,000	positive, negative, neutral	4,682	5,318	Tohoku BERT_BASE_

### 3.2 Bias evaluation measure

To comprehensively evaluate gender stereotype bias in Japanese PLMs for sentiment analysis tasks, we introduce two complementary bias detection metrics: Stereotype Bias Index (SBI) and Gender Sentiment Bias (GSB). SBI quantifies the degree of bias exhibited by the model in PS vs. AS contexts, while GSB evaluates systematic differences in the model's sentiment tendencies across male, female, and neutral contexts. To facilitate the formal definitions of these metrics and ensure clarity throughout the evaluation process, Table 2 summarizes the major mathematical notations used in this study.

**Table 2:** Major notations used.

Symbol	Description
1) Sets and models	
*c*	Sentiment class, *c*∈{Positive, Negative}.
*S*	Subset of test samples, *S*∈{PS, AS}.
*G*_*i*_, *G*_*j*_	Gender groups, e.g., {male, female, neutral}.
|*G*_*i*_|	Total number of samples in gender group *G*_*i*_.
*M*_0_	Baseline pre-trained language model before debiasing.
*M*_*d*_	Debiased sentiment analysis model after fine-tuning.
2) Basic prediction metrics	
TP_*c*_(*S*)	Number of true positives for class *c* in subset *S*.
TN_*c*_(*S*)	Number of true negatives.
FP_*c*_(*S*)	Number of false positives.
FN_*c*_(*S*)	Number of false negatives.
*R*_*c*_(*S*)	Recall for sentiment class *c* in subset *S*.
*R*_pos_(*G*_*i*_)	Average positive recall for gender group *G*_*i*_.
*R*_neg_(*G*_*i*_)	Average negative recall for gender group *G*_*i*_.
15.6-7.4,-14242pt △prob	Positive-class prediction score difference between gender-swapped sentence pairs.
3) Bias quantification metrics	
Δ_pos_	Difference in positive recall between PS and AS contexts.
Δ_neg_	Difference in negative recall between AS and PS contexts.
Δ_pos_(*G*_*i*_, *G*_*j*_)	Difference in positive recall between groups *G*_*i*_ and *G*_*j*_.
Δ_neg_(*G*_*i*_, *G*_*j*_)	Difference in negative recall between groups *G*_*i*_ and *G*_*j*_.
SBI_*B*_	Magnitude of the Stereotype Bias Index.
SBI_*D*_	Direction of the Stereotype Bias Index.
GSB_*B*_	Magnitude of Gender Sentiment Bias between gender groups.
GSB_*D*_	Direction of Gender Sentiment Bias between gender groups.

It should be noted that for datasets containing a neutral sentiment label, neutral-label samples are included when reporting general classification performance, whereas SBI and GSB are computed based on positive and negative recall differences. The SBI is designed to quantify whether a model exhibits a preference for stereotypical associations. Specifically, it measures whether the model tends to assign more positive sentiment to PS sentences compared with AS sentences, thereby indicating a bias toward PS contexts. We define the Stereotype Bias Index as follows.

** Definition 1 (Stereotype Bias Index (SBI))**. Given a sentiment class *c*∈{Positive, Negative} and a subset of test samples *S*∈{PS, AS}, the recall for class *c* in subset *S* is defined as:


$$\begin{array}{l} R_{c}\left(S\right)=\frac{\text{T}{\text{P}}_{c}\left(S\right)}{\text{T}{\text{P}}_{c}\left(S\right)+\text{F}{\text{N}}_{c}\left(S\right)}, \end{array}$$ (1)

where TP_*c*_(*S*) and FN_*c*_(*S*) denote the number of true positives and false negatives, respectively.

Based on Equation 1, the differences in recall between the pro-stereotypical (PS) and anti-stereotypical (AS) contexts for positive and negative sentiment classes are calculated as follows:


$$\begin{array}{l} {\text{Δ}}_{\text{pos}}=R_{\text{pos}}\left(\text{PS}\right)-R_{\text{pos}}\left(\text{AS}\right), \end{array}$$ (2)

$$\begin{array}{l} {\text{Δ}}_{\text{neg}}=R_{\text{neg}}\left(\text{AS}\right)-R_{\text{neg}}\left(\text{PS}\right). \end{array}$$ (3)

Using the recall differences in Equations 2 and 3, the SBI is defined by two components: the bias magnitude and the bias direction, as shown in Equations 4 and 5, respectively:


$$\begin{array}{l} {\text{SBI}}_{B}=\frac{\left|{\text{Δ}}_{\text{pos}}|+|{\text{Δ}}_{\text{neg}}\right|}{2}, \end{array}$$ (4)

$$\begin{array}{l} {\text{SBI}}_{D}=\frac{{\text{Δ}}_{\text{pos}}-{\text{Δ}}_{\text{neg}}}{2}, \end{array}$$ (5)

where SBI_*B*_ measures the magnitude of the bias, and SBI_*D*_ indicates its direction.

In the computed SBI, a value of SBI_*D*_>0 indicates that the sentiment analysis model is biased toward pro-stereotypical contexts, whereas a negative value indicates a bias toward anti-stereotypical contexts. Higher values of SBI_*B*_ correspond to a greater degree of model bias. Therefore, SBI captures the extent to which a model's sentiment predictions are influenced by gender-role stereotypes.

Because the SBI focuses on intra-gender stereotype bias by comparing PS and AS contexts within each gender group, it does not capture overall sentiment variations across different gender groups. To complement the SBI, we introduce the GSB, which measures systematic differences in sentiment predictions among male, female, and neutral contexts. The data are categorized according to gender characteristics as {male, female, neutral}, where male denotes the set of sentences containing male-related content, female denotes the set of sentences containing female-related content, and neutral denotes the set of sentences without any gender-specific content. We then formally define the Gender Sentiment Bias (GSB) as follows.

** Definition 2 (Gender Sentiment Bias (GSB))**. For each gender group *G*_*i*_∈{male, female, neutral}, the average positive and negative recalls are defined as follows:


$$\begin{array}{l} R_{\text{pos}}\left(G_{i}\right)=\frac{1}{\left|G_{i}\right|}\underset{d\in G_{i}}{\sum }\frac{\text{T}{\text{P}}_{d}}{\text{T}{\text{P}}_{d}+\text{F}{\text{N}}_{d}}, \end{array}$$ (6)

$$\begin{array}{l} R_{\text{neg}}\left(G_{i}\right)=\frac{1}{\left|G_{i}\right|}\underset{d\in G_{i}}{\sum }\frac{\text{T}{\text{N}}_{d}}{\text{T}{\text{N}}_{d}+\text{F}{\text{P}}_{d}}. \end{array}$$ (7)

Based on Equations 6 and 7, for each pair of gender groups (*G*_*i*_, *G*_*j*_), the recall differences between the two groups are computed as shown in Equations 8 and 9, respectively:


$$\begin{array}{l} {\text{Δ}}_{\text{pos}}\left(G_{i},G_{j}\right)=R_{\text{pos}}\left(G_{i}\right)-R_{\text{pos}}\left(G_{j}\right), \end{array}$$ (8)

$$\begin{array}{l} {\text{Δ}}_{\text{neg}}\left(G_{i},G_{j}\right)=R_{\text{neg}}\left(G_{i}\right)-R_{\text{neg}}\left(G_{j}\right). \end{array}$$ (9)

Finally, using the recall differences in Equations 8 and 9, the corresponding bias magnitude and direction for the gender pair (*G*_*i*_, *G*_*j*_) are formulated in Equations 10 and 11, respectively:


$$\begin{array}{l} {\text{GSB}}_{B}\left(G_{i},G_{j}\right)=\frac{\left|{\text{Δ}}_{\text{pos}}\left(G_{i},G_{j}\right)|+|{\text{Δ}}_{\text{neg}}\left(G_{i},G_{j}\right)\right|}{2}, \end{array}$$ (10)

$$\begin{array}{l} {\text{GSB}}_{D}\left(G_{i},G_{j}\right)=\frac{{\text{Δ}}_{\text{pos}}\left(G_{i},G_{j}\right)-{\text{Δ}}_{\text{neg}}\left(G_{i},G_{j}\right)}{2}. \end{array}$$ (11)

In the computed GSB metric, GSB_*B*_(*G*_*i*_, *G*_*j*_) quantifies the overall deviation in sentiment predictions between gender contexts *G*_*i*_ and *G*_*j*_. A larger value indicates that the model is more sensitive to gender-based differences. The bias direction GSB_*D*_(*G*_*i*_, *G*_*j*_) characterizes the system's polarity tendency under different gender contexts. When GSB_*D*_(*G*_*i*_, *G*_*j*_)>0, sentences involving *G*_*i*_ are more likely to be predicted as positive. When GSB_*D*_(*G*_*i*_, *G*_*j*_) < 0, sentences involving *G*_*j*_ are more likely to receive positive predictions. In the overall framework, we employ both SBI and GSB to evaluate the degree of gender bias in the PLMs (e.g., denoted as model *M*_0_) as well as in the models after debiasing fine-tuning. The use of recall-based differences is appropriate for this study because recall measures whether samples of a given sentiment class are correctly recognized under different gender-related contexts. Compared with overall accuracy, recall is more sensitive to class-specific disparities and can reveal cases where a model maintains high aggregate performance but recognizes positive or negative sentiment unevenly across PS, AS, male, female, or neutral contexts. Thus, SBI and GSB provide prediction-level and class-sensitive measures for examining whether gender cues affect sentiment analysis behavior.

### 3.3 Construction of the debiased training data

After identifying the presence of gender-stereotypical bias in sentiment analysis models, we further construct a debiased training data using a gender-swap strategy and fine-tune the models on this modified data. This approach has been widely used to reduce explicit gender cues in text while preserving the original semantic meaning of sentences Anantaprayoon et al. (2024). By systematically removing or replacing gender markers, the model is encouraged to learn sentiment associations that are independent of gender, thereby mitigating spurious correlations between gendered terms and sentiment labels.

#### 3.3.1 Original data selection

In the original corpus, we first extract sentences containing explicit gender references, including first-person pronouns (eg., 私, 僕, 俺) and third-person pronouns (e.g., 彼, 彼女). We then retain only those sentences that also include occupation- or role-related terms, or descriptive content such as adjectives or activities, to ensure that the resulting subset covers contexts with substantive semantic meaning. This enables more effective capture of the potential associations between gender cues and sentiment expressions. The selection process was implemented using predefined lexical rules, which specified gender terms, pronouns, self-reference forms, occupation-related terms, role nouns, adjectives, and activity-related descriptions used to identify candidate sentences.

#### 3.3.2 Gender-swapping procedure

All selected sentences contain explicit gender references. For these sentences, we perform gender swapping by systematically replacing gender-related terms with their corresponding alternatives, such as 彼女 → 彼, 女性 → 男性, 母親 → 父親. During this process, necessary syntactic adjustments, such as verb conjugations and particle modifications, are applied to ensure the grammatical correctness of the generated sentences. For each original sentence, we generate a pair of gender-swapped variants, thereby achieving balanced representation across different gender categories. At the same time, the original sentiment labels (positive, negative, or neutral) are retained, ensuring that the semantic content remains intact while decoupling gender information from the target labels.

The resulting debiased training dataset maintains semantic consistency, neutralizes gender-sentiment associations, and provides a reliable foundation for fine-tuning PLMs toward unbiased predictions. Because Japanese gender expressions may involve pragmatic and stylistic nuances, this substitution-based strategy should be interpreted as a simple data-centric intervention rather than a full treatment of all sociolinguistic forms of gendered meaning in Japanese.

### 3.4 Systematic pipeline for bias detection and debiasing

**Figure 2:**
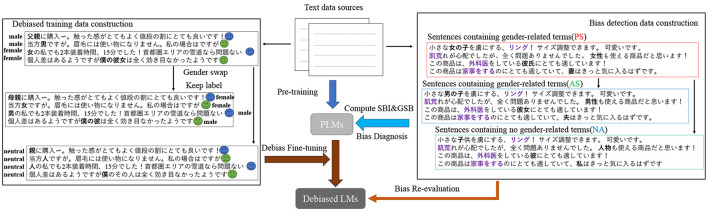
The overall framework of the bias detection and debiasing in Japanese PLM for sentiment analysis tasks, consisting of three stages: bias diagnosis, debiasing fine-tuning, and bias re-evaluation.

Building on the aforementioned procedures for constructing bias detection data, bias evaluation measure, and constructing the debiased training data, we propose a systematic three-stage framework for diagnosing, mitigating, and evaluating gender-stereotypical bias in Japanese PLMs for sentiment analysis tasks. The systematic framework is shown in Figure 2. In the debiasing training data construction shown in Figure 2, the smiling-face icon represents positive labels, the crying-face icon represents negative labels, and the bold text highlights gendered or gender-neutral terms. In the the test data construction shown in Figure 2, Japanese words in purple are used to emphasize semantic elements associated with gender bias, such as: 外科医 (a profession linked to gender stereotypes), 肌荒れ (commonly appearing in products targeted at specific genders), 家事をする (a household activity traditionally associated with women), and リング (a product with gendered connotations). The framework provides a unified methodology for systematically assessing and reducing the sensitivity of pretrained models to gender-related cues.

Our framework consists of three sequential stages: Bias Diagnosis, Debias Fine-tuning, and Bias Re-evaluation. In the bias diagnosis stage, the constructed bias detection data along with the SBI and GSB metrics are used to quantify gender bias in the PLMs. In the debias fine-tuning stage, gender-related bias is mitigated by fine-tuning the models on the constructed debiased training data. In the bias re-evaluation stage, the same bias detection data are used to re-quantify the bias levels of the fine-tuned models, thereby validating the effectiveness of the debiasing strategy.

#### 3.4.1 Stage 1: bias diagnosis

In the first stage, we diagnose the baseline model *M*_0_, which is a Japanese PLM for sentiment analysis tasks, using the constructed PS, AS, and NA test sets. Depending on the dataset and model, the PLM performs either binary sentiment prediction (positive and negative) or three-class sentiment prediction (positive, negative, and neutral). The model is assessed using standard performance metrics as well as our proposed bias quantification measures, SBI and GSB, which capture the model's predictive performance and bias level, respectively. For consistency across binary and three-class settings, SBI and GSB are computed based on positive and negative recall differences, while the neutral sentiment class, when present, is retained in the general performance evaluation. For the baseline model *M*_0_, the bias values computed using the SBI metric are denoted as ${\text{SBI}}_{B}^{M_{0}}$ and ${\text{SBI}}_{D}^{M_{0}}$, while the bias values computed using the GSB metric are denoted as ${\text{GSB}}_{B}^{M_{0}}$ and ${\text{GSB}}_{D}^{M_{0}}$. Observing results such as ${\text{SBI}}_{B}^{M_{0}}\approx 0.02$ or ${\text{SBI}}_{D}^{M_{0}}>0$ indicates that the PLM exhibits measurable gender-stereotypical tendencies, highlighting the necessity of applying debiasing interventions to this model.

#### 3.4.2 Stage 2: debias fine-tuning

To mitigate the gender bias identified in the first stage, we fine-tune the model on the debiased training data constructed via systematic gender swapping. The fine-tuned model is initialized with the parameters of *M*_0_ and trained under the original sentiment classification objective. During each training epoch, for every original sentence pair, one gender-swapped variant is randomly selected (50% male and 50% female) and included in the training batch. This sampling strategy ensures that the model cannot rely on gender cues for prediction. As a result, the resulting model *M*_*d*_ exhibits substantially reduced gender-related sentimental bias.

#### 3.4.3 Stage 3: bias re-evaluation

In this stage, we re-evaluate the debiased model *M*_*d*_ using the same bias detection data as in Stage 1, and quantify its gender bias using the SBI and GSB metrics. For the debiased model *M*_*d*_, the bias values computed using the SBI metric are denoted as ${\text{SBI}}_{B}^{M_{d}}$ and ${\text{SBI}}_{D}^{M_{d}}$, while the bias values computed using the GSB metric are denoted as ${\text{GSB}}_{B}^{M_{d}}$ and ${\text{GSB}}_{D}^{M_{d}}$. We assess the effectiveness of the fine-tuning process in reducing stereotypical bias and gender-related sentiment tendencies by directly comparing the bias values of *M*_0_ and *M*_*d*_. A decrease in bias values indicates that gender bias has been successfully mitigated, ensuring that the observed improvements stem from the debiasing strategy itself rather than differences in data distribution or evaluation conditions.

By integrating the three stages described above, this study establishes a transparent and model-agnostic framework for diagnosing and mitigating gender-stereotypical bias in Japanese PLMs for sentiment analysis tasks.

## 4 Experiments and analyses

### 4.1 Japanese sentiment analysis models

In this study, we conduct experiments based on three widely used Japanese PLMs to evaluate the effectiveness of the proposed systematic framework for bias quantification and debiasing. The three PLMs are Tohoku BERT_BASE_^1^, Tohoku BERT_BASE_ (chABSA)^2^, and Tohoku BERT_BASE_ (JSPD)^3^. Each model is pre-trained on a different corpus and subsequently fine-tuned for Japanese sentiment analysis tasks. All models adopted an architecture consistent with the original BERT, comprising 12 encoder layers, 768-dimensional hidden states, and 12 attention heads. Table 3 summarizes the emotion classification settings of the three sentiment analysis models described above, as well as the training corpora used for each model.

**Table 3:** The description of Japanese PLMs for sentiment analysis tasks.

Model	Classes	Pre-trained dataset
Tohoku BERT_BASE_	positive, negative, neutral	Japanese version of Wikipedia
Tohoku BERT_BASE_ (chABSA)	positive, negative	Aspect based sentiment analysis in Japanese
Tohoku BERT_BASE_ (JSPD)	positive, negative	Japanese sentiment polarity dictionary

### 4.2 Datasets

For the three PLMs mentioned above, we employ two datasets for bias quantification and debiasing training: the Japanese Sentiment Classification (JSC) dataset and the Multilingual Amazon Reviews (MAR) dataset. The JSC dataset is a subset of the JMTEB benchmark^4^ specifically designed for sentiment analysis. It contains positive and negative sentiment labels and is therefore used to train the Tohoku BERT_BASE_ (chABSA) and Tohoku BERT_BASE_ (JSPD) models. The MAR dataset is sourced from the Multilingual Sentiment Datasets^5^, a collection that integrates user reviews in multiple languages from various platforms. Specifically, MAR includes Japanese customer reviews from Amazon, annotated with three sentiment categories: positive, negative, and neutral, and is used to train the Tohoku BERT_BASE_ model. Table 1 summarizes the statistics of the JSC and MAR datasets, the bias detection tasks for which they are used by different models, and the sample sizes of the bias detection data constructed from these datasets. It should be noted that JSC and MAR differ in domain, label structure, and dataset size. Therefore, the experimental results are not intended to support direct cross-dataset or cross-model ranking. Instead, the main evaluation principle of this study is within-model comparison: for each PLM, the bias scores and classification performance before and after debiasing are compared using the same dataset, the same bias detection data, and the same evaluation protocol. The results from different models are reported together for compact presentation, but the effectiveness of debiasing is assessed primarily according to the before-and-after changes within each model.

### 4.3 Experimental details

For the debiasing fine-tuning of PLMs, we employed the AdamW optimizer, coupled with a cosine learning rate decay schedule with warmup. For all experiments, we used a learning rate of 2 × 10^−5^, a weight decay of 0.01, a batch size of 32, and a maximum of 5 training epochs. The training process incorporated an early stopping mechanism, which was triggered when the Macro-F1 score on the development set ceased to improve. To reduce the influence of random initialization and data-order variation, all experiments were repeated using five random seeds: 1, 24, 42, 123, and 2024. The reported results are presented as the mean ± standard deviation across these five runs. The evaluation metrics for the sentiment analysis models were categorized into two groups: performance evaluation and bias quantification. For performance evaluation, we employed Accuracy (ACC), F1-score (F1), and Recall. For bias quantification, we utilized the two metrics proposed in this work: the SBI and GSB.

### 4.4 Experimental results

#### 4.4.1 Quantitative analysis of debiasing effects

Table 4 summarizes the performance and SBI scores of the three Japanese PLMs for sentiment analysis tasks before debiasing. Table 5 reports the corresponding GSB scores across different gender-group comparisons before debiasing. Tables 6, 7 further compare the proposed method with Gender-Inclusive Language Fine-Tuning (GIL-FT). GIL-FT is a data-centric debiasing baseline that fine-tunes models using gender-inclusive data, in which gender-exclusive or gender-marked expressions are replaced with more gender-neutral alternatives (Bartl and Leavy, 2024). This comparison allows us to examine whether our gender-swapped fine-tuning strategy can more effectively reduce SBI and GSB while preserving sentiment classification performance.

**Table 4:** Bias score (SBI) and classification performance of each PLM before debiasing.

Model	Subset	Accuracy	F1	Recall	SBI_*B*_	SBI_*D*_
Tohoku BERT_BASE_	PS	0.9428	0.9606	0.9630		
	AS	0.9096	0.9355	0.9089	0.038	0.016
	NA	0.9197	0.9353	0.9451		
Tohoku BERT_BASE_(chABSA)	PS	0.9520	0.9689	0.9981		
	AS	0.9364	0.9564	0.9658	0.040	-0.007
	NA	0.9211	0.9368	0.9508		
Tohoku BERT_BASE_(JSPD)	PS	0.7342	0.6937	0.6776		
	AS	0.7190	0.6720	0.6584	0.013	0.013
	NA	0.7217	0.7091	0.7016		

**Table 5:** GSB scores of each PLM before debiasing.

Model	Comparison	GSB_*B*_	GSB_*D*_
Tohoku BERT_BASE_	Male vs. Female	0.033	0.033
	Male vs. Neutral	0.015	–0.001
	Female vs. Neutral	0.033	–0.033
Tohoku BERT_BASE_(chABSA)	Male vs. Female	0.041	0.041
	Male vs. Neutral	0.055	0.055
	Female vs. Neutral	0.014	0.014
Tohoku BERT_BASE_(JSPD)	Male vs. Female	0.024	–0.021
	Male vs. Neutral	0.064	0.064
	Female vs. Neutral	0.086	0.084

**Table 6:** Comparison of bias score (SBI) and classification performance of different debiasing methods after fine-tuning.

Model	Method	Subset	Accuracy	F1	Recall	SBI_*B*_	SBI_*D*_
Tohoku BERT_BASE_	GIL-FT	PS	0.9428 ± 0.0176	0.9278 ± 0.0110	0.9248 ± 0.0103		
		AS	0.9343 ± 0.0148	0.9191 ± 0.0114	0.9261 ± 0.0113	0.0097 ± 0.0027	0.0097 ± 0.0027
		NA	0.9200 ± 0.0091	0.9149 ± 0.0063	0.9119 ± 0.0050		
	Ours	PS	0.9668 ± 0.0034	0.9779 ± 0.0049	0.9990 ± 0.0092		
		AS	0.9647 ± 0.0090	0.9764 ± 0.0095	0.9962 ± 0.0074	0.0015 ± 0.0003	0.0015 ± 0.0003
		NA	0.9203 ± 0.0043	0.9373 ± 0.0032	0.9676 ± 0.0027		
Tohoku BERT_BASE_(chABSA)	GIL-FT	PS	0.9594 ± 0.0012	0.9566 ± 0.0016	0.9808 ± 0.0022		
		AS	0.9594 ± 0.0012	0.9566 ± 0.0016	0.9808 ± 0.0022	0.0126 ± 0.0067	0.0126 ± 0.0067
		NA	0.9220 ± 0.0006	0.9163 ± 0.0010	0.9102 ± 0.0027		
	Ours	PS	0.9661 ± 0.0012	0.9774 ± 0.0015	0.9981 ± 0.0022		
		AS	0.9534 ± 0.0012	0.9692 ± 0.0015	0.9811 ± 0.0022	0.0116 ± 0.0067	0.0116 ± 0.0067
		NA	0.9200 ± 0.0008	0.9370 ± 0.0009	0.9676 ± 0.0009		
Tohoku BERT_BASE_(JSPD)	GIL-FT	PS	0.7412 ± 0.0176	0.6946 ± 0.0271	0.6880 ± 0.0319		
		AS	0.7333 ± 0.0189	0.6870 ± 0.0234	0.6801 ± 0.0267	0.0058 ± 0.0003	0.0058 ± 0.0006
		NA	0.7137 ± 0.0173	0.7136 ± 0.0167	0.7097 ± 0.0203		
	Ours	PS	0.7909 ± 0.0019	0.7711 ± 0.0063	0.7661 ± 0.0054		
		AS	0.7874 ± 0.0059	0.7679 ± 0.0167	0.7631 ± 0.0151	0.0029 ± 0.0008	0.0018 ± 0.0008
		NA	0.7809 ± 0.0018	0.7740 ± 0.0019	0.7725 ± 0.0019		

Results are reported as mean ± standard deviation over five random seeds.

**Table 7:** Comparison of GSB scores of different debiasing methods after fine-tuning.

Model	Method	Comparison	GSB_*B*_	GSB_*D*_
Tohoku BERT_BASE_	GIL-FT	Male vs. Female	0.0198 ± 0.0114	0.0171 ± 0.0010
		Male vs. Neutral	0.0206 ± 0.0012	0.0015 ± 0.0011
		Female vs. Neutral	0.0298 ± 0.0025	0.0106 ± 0.0141
	Ours	Male vs. Female	0.0080 ± 0.0022	0.0080 ± 0.0022
		Male vs. Neutral	0.0101 ± 0.0022	0.0030 ± 0.0017
		Female vs. Neutral	0.0050 ± 0.0055	−0.0050 ± 0.0055
Tohoku BERT_BASE_(chABSA)	GIL-FT	Male vs. Female	0.0315 ± 0.0063	0.0235 ± 0.0095
		Male vs. Neutral	0.0150 ± 0.0001	0.0042 ± 0.0207
		Female vs. Neutral	0.0099 ± 0.0002	0.0077 ± 0.0029
	Ours	Male vs. Female	0.0028 ± 0.0025	0.0028 ± 0.0025
		Male vs. Neutral	0.0037 ± 0.0009	0.0037 ± 0.0026
		Female vs. Neutral	0.0065 ± 0.0009	0.0065 ± 0.0003
Tohoku BERT_BASE_(JSPD)	GIL-FT	Male vs. Female	0.0121 ± 0.0180	0.01042 ± 0.0180
		Male vs. Neutral	0.0454 ± 0.0027	−0.0336 ± 0.0093
		Female vs. Neutral	0.0737 ± 0.0107	0.0706 ± 0.0107
	Ours	Male vs. Female	0.0031 ± 0.0013	0.0031 ± 0.0004
		Male vs. Neutral	0.0042 ± 0.0019	0.0042 ± 0.0005
		Female vs. Neutral	0.0011 ± 0.0010	0.0011 ± 0.0005

Results are reported as mean ± standard deviation over five random seeds.

Because the models are associated with different datasets, these tables should be interpreted primarily as within-model before-and-after comparisons rather than direct cross-model comparisons. As shown in Table 4, all three models exhibit a clear performance gap between the PS and AS subsets before debiasing, with consistently higher accuracy, F1 scores, and recall on the PS subset. This systematic disparity results in elevated SBI_*B*_ values, indicating that the models tend to associate gender-related contexts with specific sentiment polarities. Despite being extensively trained for sentiment inference, the models still display measurable gender-related bias, and the degree of such bias varies across models. Among the three models, Tohoku BERT_BASE_ and Tohoku BERT_BASE_(chABSA) exhibit higher SBI_*B*_ values, whereas Tohoku BERT_BASE_(JSPD) shows a comparatively smaller performance difference between the PS and AS subsets. This suggests that stereotype-level bias differs across model and dataset settings. The GSB results in Table 5 further show that gender-group sentiment disparities exist across male, female, and neutral comparisons, although the largest GSB_*B*_ values appear in different gender pairs for different models. Therefore, the GSB results should be interpreted as evidence of gender-sensitive prediction behavior rather than as a direct ranking of the three models.

After debiasing, the proposed gender-swapping strategy substantially reduces stereotype-level bias. As shown in Table 6, the SBI_*B*_ values of Tohoku BERT_BASE_, Tohoku BERT_BASE_(chABSA), and Tohoku BERT_BASE_(JSPD) decrease from 0.038 to 0.0015, from 0.040 to 0.0116, and from 0.013 to 0.0029, corresponding to reductions of 96.1%, 71.0%, and 77.7%, respectively. These results confirm that the proposed method effectively mitigates PS–AS stereotype-level bias. In terms of SBI_*D*_, the directional biases of Tohoku BERT_BASE_ and Tohoku BERT_BASE_(JSPD) are greatly reduced and approach zero. For Tohoku BERT_BASE_(chABSA), the sign of SBI_*D*_ changes after debiasing, indicating a slight reversal of directional bias; nevertheless, the overall bias magnitude measured by SBI_*B*_ is still substantially reduced. The GSB results in Table 7 further demonstrate that the proposed method reduces gender-group sentiment bias. Compared with the before-debiasing results in Table 5, the proposed method reduces GSB_*B*_ across all gender-group comparisons. Notably, for Tohoku BERT_BASE_(JSPD), the GSB_*B*_ values for male vs. female, male vs. neutral, and female vs. neutral decrease from 0.024 to 0.0031, from 0.064 to 0.0042, and from 0.086 to 0.0011, corresponding to reductions of 87.1%, 93.4%, and 98.7%, respectively. These reductions indicate that gender-related sentiment disparities are substantially attenuated after debiasing. The corresponding GSB_*D*_ values are also close to zero in most cases, suggesting that the proposed method does not introduce strong new directional gender bias.

Compared with GIL-FT, the proposed method achieves lower SBI_*B*_ values for all three models. Specifically, for Tohoku BERT_BASE_, Tohoku BERT_BASE_(chABSA), and Tohoku BERT_BASE_(JSPD), the SBI_*B*_ values decrease from 0.0097, 0.0126, and 0.0058 under GIL-FT to 0.0015, 0.0116, and 0.0029 under the proposed method, respectively. The advantage is also evident for GSB: across all models and gender-pair comparisons, the proposed method obtains lower GSB_*B*_ values than GIL-FT. For example, for Tohoku BERT_BASE_(chABSA), the proposed method achieves GSB_*B*_ values of 0.0028, 0.0037, and 0.0065 for the male vs. female, male vs. neutral, and female vs. neutral pairs, whereas GIL-FT obtains 0.0315, 0.0150, and 0.0099, respectively. These results show that the proposed gender-swapped fine-tuning strategy provides stronger and more consistent bias reduction than GIL-FT. More importantly, the proposed method generally maintains or improves sentiment classification performance. Compared with GIL-FT, it achieves higher F1 scores and recall values in most PS, AS, and NA subsets. Although a small decrease in accuracy is observed for some subsets of Tohoku BERT_BASE_(chABSA), the overall classification performance remains stable while the bias scores are further reduced. This indicates that the proposed method achieves a better balance between bias mitigation and task performance.

Overall, these results demonstrate that Japanese PLMs encode detectable gender-stereotypical and gender-group sentiment biases, but such biases can be effectively mitigated through targeted gender-swapped fine-tuning. Compared with GIL-FT, the proposed method provides stronger reductions in both SBI and GSB while preserving the sentiment classification capability of the models.

#### 4.4.2 Qualitative analysis of debiasing effects

**Figure 3:**
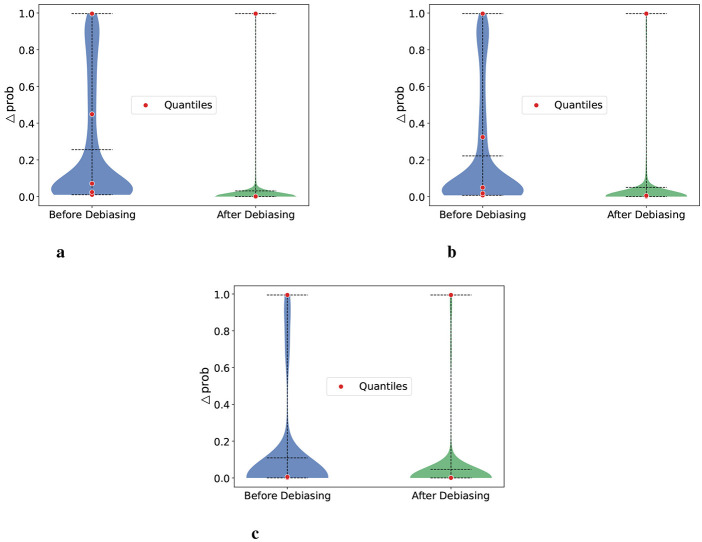
Distributional differences in △prob for gender-swapped sentences before and after debiasing across three PLMs. Red points denote sample quantiles (0%, 25%, 50%, 75%, and 100%), while the dashed line indicates the mean △prob. **(a)** Tohoku BERT_BASE_. **(b)** Tohoku BERT_BASE_(chABSA). **(c)** Tohoku BERT_BASE_(JSPD).

Figure 3 presents violin plots visualizing the distribution of the positive-class prediction score differences (△prob) between gender-swapped sentence pairs (PS and AS) before and after debiasing across the three sentiment analysis models. Before debiasing, the quantile distributions of all three models are broad and asymmetric, typically deviating substantially from zero, an effect most pronounced in the Tohoku BERT_BASE_ and Tohoku BERT_BASE_ (chABSA) models. This dispersion indicates that gender substitution induces notable fluctuations in predicted probabilities, suggesting that the models rely on gender-related lexical cues when making sentiment predictions. Meanwhile, the mean values for all three models deviate clearly from zero, reflecting not only high variance but also systematic directional bias in the prediction shifts.

After applying the debiasing strategy, the quantile distributions become markedly narrower. The red points cluster closer to zero, and the violin plots become slimmer and more symmetric, indicating a substantial reduction in prediction differences between gender-swapped inputs. This suggests that the models respond far more consistently when gender information is altered. The decreased quantile range further demonstrates that extreme probability deviations, which were previously common in the original models, have largely disappeared. Moreover, after debiasing, the mean of △prob for all three models shifts toward zero and remains centered. This shift not only reflects reduced variability but also indicates that the debiasing process effectively removes the systematic component of gender bias in the predictions.

These visualization results are highly consistent with the quantitative metrics reported earlier, further confirming the robustness and generalizability of the debiasing strategy across different models and datasets.

### 4.5 Discussion

The comprehensive quantitative and qualitative analyses yield several critical insights into the behavior of Japanese PLMs. The analysis of the bias metrics (SBI and GSB) reveals that all evaluated models exhibit clear and quantifiable sensitivity to gender cues, both at the stereotype level and at the broader level of gender attributes. Specifically, the PLMs tend to produce more positive sentiment predictions for contexts aligned with social gender stereotypes, and they display asymmetric bias when those stereotypes are violated. This trend is highly consistent with previous findings in English and other languages, suggesting that gender stereotypes embedded in training corpora naturally transfer to downstream tasks. Notably, this transfer occurs even in Japanese, where gender marking is relatively implicit compared to morphologically rich languages, underscoring the subtle yet pervasive nature of representational harms. More importantly, these findings indicate that gender bias in Japanese PLMs is not only a representation-level phenomenon, but can also emerge in task-level sentiment prediction. In other words, gender-related cues may affect whether positive or negative sentiment is correctly recognized, even when the core sentiment semantics remain comparable. This supports the need for prediction-level bias evaluation in sentiment analysis, rather than relying solely on intrinsic measures such as embedding association or token-level probability differences.

From a methodological perspective, the proposed pipeline evaluates a specific and operational form of fairness: whether sentiment analysis models respond consistently to stereotype-aligned, stereotype-violating, and gender-neutral contexts. SBI captures stereotype-level disparities between PS and AS samples, while GSB captures broader sentiment differences across male, female, and neutral groups. These two metrics complement each other by distinguishing whether bias arises from stereotype alignment, general gender-group sentiment imbalance, or both.

The comparison with Gender-Inclusive Language Fine-Tuning (GIL-FT) further highlights the effectiveness of the proposed strategy. While GIL-FT mitigates bias by replacing gender-exclusive or gender-marked expressions with more gender-neutral alternatives, our method explicitly constructs gender-swapped training data aligned with the sentiment analysis setting. As a result, the proposed method achieves lower SBI_*B*_ values than GIL-FT across all three PLMs and also yields consistently lower GSB_*B*_ values across gender-pair comparisons. This suggests that task-oriented gender swapping is more effective in weakening stereotype-level and gender-group sentiment bias, while still preserving overall sentiment classification performance.

Furthermore, our results confirm that fairness-oriented data interventions can be seamlessly integrated into real-world Japanese sentiment analysis models, neutralizing spurious correlations without sacrificing predictive accuracy. At the same time, the proposed intervention should be interpreted as a data-centric mitigation strategy rather than a complete solution to gender bias. Gender-swapped fine-tuning can reduce the model's reliance on explicit gender markers and weaken spurious gender–sentiment associations, but it cannot fully address more implicit pragmatic, stylistic, or sociolinguistic forms of gendered meaning in Japanese. Therefore, the proposed framework provides a transparent task-level evaluation and mitigation pipeline, while leaving more complex forms of Japanese gender expression for future investigation.

## 5 Conclusion

In this paper, we propose a systematic framework for diagnosing, mitigating, and evaluating gender-stereotypical bias in Japanese pre-trained language models applied to sentiment analysis tasks. We constructed a bias detection dataset incorporating stereotype-aligned principles and introduced two complementary metrics, SBI and GSB, for a multidimensional assessment of model fairness. By applying a gender-swapping strategy for debiasing fine-tuning, our findings confirm that while Japanese PLMs inherently encode detectable gender-related patterns from their pre-training corpora, the proposed intervention effectively suppresses these directional biases. Crucially, this debiasing is achieved while preserving-and in some cases slightly enhancing-downstream sentiment classification capabilities.

This study advances both the methodological and empirical understanding of gender-stereotypical bias in Japanese NLP. While the proposed framework demonstrates high efficacy, it primarily focuses on sentence-level sentiment classification and foundational gender expressions, not fully encapsulating rich sociolinguistic complexities such as gendered honorifics (Keigo) or dialectal variations. Future research should therefore extend this methodology to more nuanced linguistic phenomena and broader affective computing tasks, such as document-level sentiment analysis and multi-label emotion recognition.

## Data Availability

Publicly available datasets were analyzed in this study. This data can be found here: The datasets used for this study consist exclusively of publicly available, non-identifiable text corpora. The Japanese Sentiment Classification dataset (JSC) is publicly accessible via the JMTEB benchmark at https://huggingface.co/datasets/sbintuitions/JMTEB. The Multilingual Amazon Reviews (MAR) dataset is publicly available at https://huggingface.co/datasets/tyqiangz/multilingual-sentiments. All gender-swapped data used for debiasing were automatically generated from these public datasets and contain no personal, private, or identifiable human information. The processed datasets and debiasing scripts are available from the corresponding author upon reasonable request.

## Footnotes

1
https://huggingface.co/tohoku-nlp/bert-base-japanese-v2

2
https://huggingface.co/jarvisx17/japanese-sentiment-analysis

3
https://huggingface.co/minutillamolinara/bert-japanese_finetuned-sentiment-analysis

4
https://huggingface.co/datasets/sbintuitions/JMTEB

5
https://huggingface.co/datasets/tyqiangz/multilingual-sentiments
